# Most parsimonious haplotype allele sharing determination

**DOI:** 10.1186/1471-2105-10-115

**Published:** 2009-04-21

**Authors:** Zhipeng Cai, Hadi Sabaa, Yining Wang, Randy Goebel, Zhiquan Wang, Jiaofen Xu, Paul Stothard, Guohui Lin

**Affiliations:** 1 Department of Computing Science, University of Alberta, Edmonton, Alberta, T6G 2E8, Canada; 2 Department of Agriculture, Food, and Nutritional Science, University of Alberta, Edmonton, Alberta, T6G 2P5, Canada

## Abstract

**Background:**

The "common disease – common variant" hypothesis and genome-wide association studies have achieved numerous successes in the last three years, particularly in genetic mapping in human diseases. Nevertheless, the power of the association study methods are still low, in particular on quantitative traits, and the description of the full allelic spectrum is deemed still far from reach. Given increasing density of single nucleotide polymorphisms available and suggested by the block-like structure of the human genome, a popular and prosperous strategy is to use haplotypes to try to capture the correlation structure of SNPs in regions of little recombination. The key to the success of this strategy is thus the ability to unambiguously determine the haplotype allele sharing status among the members. The association studies based on haplotype sharing status would have significantly reduced degrees of freedom and be able to capture the combined effects of tightly linked causal variants.

**Results:**

For pedigree genotype datasets of medium density of SNPs, we present two methods for haplotype allele sharing status determination among the pedigree members. Extensive simulation study showed that both methods performed nearly perfectly on breakpoint discovery, mutation haplotype allele discovery, and shared chromosomal region discovery.

**Conclusion:**

For pedigree genotype datasets, the haplotype allele sharing status among the members can be deterministically, efficiently, and accurately determined, even for very small pedigrees. Given their excellent performance, the presented haplotype allele sharing status determination programs can be useful in many downstream applications including haplotype based association studies.

## Background

With the completion of the Human Genome Project, coordinated effort has made available millions of single nucleotide polymorphisms (SNPs). These SNPs represent many of the genetic variants in the human genome and they will greatly facilitate the identification of genetic variants underlying human diseases, the goal of association studies. Indeed, under the "common disease – common variant (CD-CV)" hypothesis, genome-wide association studies (GWASs) have achieved numerous successes in the last three years, particularly in genetic mapping in human diseases [1]. For example, reproducible associations have been described for many human common conditions and diseases, such as obesity [2], diabetes [3], and rheumatoid arthritis [4]. The genetic markers revealed by these studies may provide insights into the underlying molecular pathways, and may lead to novel strategies for disease diagnosis, treatment, and prevention.

In general, there are three different kinds of association studies: case-control, categorical disease outcomes, and quantitative (continuous). Each association study may deal with only a single SNP and multiple SNPs. It appears that the first two kinds of association studies are much easier than the quantitative ones, and in fact the past successes are all on the first two kinds. For the last kind, most association study methods are regression based, and they become either ineffective (undesirable results) or inefficient (exceptionally long computational time) with increasing numbers of SNPs [5]. It is recognized that, despite the many achieved successes, the power of the association study methods are nevertheless still low, and there remain many more important diseases to be studied, particularly quantitative ones.

The major issue in the current technical themes of GWASs is the data dimensionality, where the number of samples is far less than the number of genotyped SNPs. This issue becomes particularly severe when dealing with rare diseases. Impacted by linkage disequilibrium (LD), and that the human genome can be partitioned into large blocks with high LD and relatively low recombination (or crossover, or breakpoint), separated by short regions of low LD [6-8], SNP tagging has been proposed to reduce the number of SNPs to the minimum while retaining as much the genetic variation of the full SNP set as possible. However, in practice, tagging is only effective in capturing common variants. A popular and prosperous strategy, suggested by this block-like structure of the human genome, is to use haplotypes to try to capture the correlation structure of SNPs in regions of little recombination [9-11]. This approach can lead to analyses with significantly reduced degrees of freedom and, more importantly, haplotypes are able to capture the combined effects of tightly linked causal variants.

Haplotypes are very expensive to assay. For the vast majority of applications that involve large numbers of samples, only genotype data are available through high-throughput genotyping technologies. One of the potential problems underlying the haplotype-based association study methods is that haplotypes are not observed but rather inferred, and it can be difficult to account for the uncertainty that arises in phase inference when assessing the overall significance of the association. One solution to this problem is to determine the haplotype allele sharing status among *all *members in the study [10,12]. In particularly, it is expected that the availability of high density SNP genotype data can be used to unambiguously determine the haplotype allele sharing.

In this work, we demonstrate that, for pedigree genotype datasets, such haplotype allele sharing can indeed be deterministically, efficiently, and accurately determined, even for very small pedigrees. This confirms that haplotype-based association studies are promising for flexible and interpretable analyses that exploit evolutionary insights.

We determine the haplotype allele sharing status among pedigree members using two distinct parsimonious haplotyping algorithms with different optimization objective functions: one minimizes the total number of crossovers to explain the pedigree genotype data by Mendelian inheritance rules; the other minimizes the total number of crossover sites, or equivalently, minimizes the number of maximal zero-recombination chromosomal regions. Both haplotyping results give unambiguous haplotype allele sharing status among the members, as well as the shared haplotype alleles, though the phases for each individual might not be completely determined. These shared haplotype alleles can provide additional support for mapping phenotype genes [13-19], and they may also lead to insights on the factors influencing the dependencies amongst genetic markers, *i.e*. linkage disequilibrium. Such insights may prove essential to understanding genome evolution [20,21] (see the International HapMap Project [22]).

There is a rich and growing literature on haplotype inference, or *haplotyping*. Some works focus on unrelated individuals [23-30]; Research on related individuals include those based on either exact-likelihood computations [31-34], approximate-likelihood computations [31,32,35], or rule-based strategies [12,36-41]. Conceivably, all of these haplotyping algorithms, methods and programs have elements in common and have their own strengths and weaknesses. For example, the likelihood-based methods are in general limited to a small number of markers and small pedigrees, owing to the extensive computations required. Additional information and assumptions, such as marker recombination rates and Hardy-Weinberg equilibrium, are generally required to calculate the likelihood. The rule-based methods are *ad hoc *but they rely on fewer assumptions and generally run faster than likelihood-based methods. Most recently, Lin *et al*. developed a greedy haplotyping algorithm that takes advantage of the high density SNP markers [12]. This algorithm is incorporated into the *i *Linker program, which determines the haplotype allele sharing among pedigree members. This method will be used in this study. Essentially, *i *Linker uses a minimum number of breakpoints to explain the genotype data, in the presence of missing genotypes and genotype errors. Then, during the data interpretation process, parental haplotype phases are revised when more members are added, as long as the revision reduces the total number of breakpoints and still explains the genotype data. The substantial simulation study in [12] has previously demonstrated its efficiency, effectiveness, and reliability.

The computational problem of finding an optimal haplotype configuration (i.e., containing a minimum number of breakpoints) for a pedigree genotype dataset is in general NP-hard [42]. That is, there is unlikely a polynomial time algorithm that guarantees reconstruction of an optimal haplotype configuration for any pedigree genotype dataset. When no recombination events are allowed, the haplotype inference becomes easy. In the absence of missing genotype data, Li and Jiang [42] proposed a polynomial time exact algorithm for this *zero-recombination haplotype configuration *(ZRHC) problem, to reconstruct all compatible haplotype configurations without recombination. This algorithm, implemented within PedPhase [42], runs in *O*(*m*^3^*n*^3^) time, where *m *is the number of SNPs under consideration and *n *is the number of non-founder members in the pedigree. Following several major advances [43,44], Liu and Jiang [45] recently designed an *O*(*mn*) time algorithm which generates a particular solution to the ZRHC problem and, in *O*(*mn*^2^) time, it generates a general solution (or all solutions). Other works on the ZRHC problem include Cox *et al*. [46], Haplore by Zhang *et al*. [47], and ZRHI by Wang *et al*. [48]. It should be mentioned that one immediate application of ZRHC is the association studies that involve many tightly linked markers in a small chromosomal region. Within such a small region, recombination is an unlikely event and thus it is reasonable to assume that there is no recombination among these markers across the pedigrees studied [8].

In this work, we extend the PedPhase zero-recombination haplotyping algorithm to xPedPhase to determine all *maximal zero-recombination chromosomal regions*, as well as their respective haplotype configurations, in one whole genome scan. Subsequently, the haplotype allele sharing status among the pedigree members at each maximal zero-recombination chromosomal region can be determined. Note that it is infeasible to assume *no *crossover events for a whole chromosome. One may run the original PedPhase multiple times on all possible chromosomal intervals to identify the maximal zero-recombination chromosomal regions. However, this would take a prohibitive amount of time as PedPhase runs in cubic time. Therefore, our extension xPedPhase to determine *all *maximal zero-recombination chromosomal regions in one whole genome scan is non-trivial. We note that this way of haplotype allele sharing determination differs from that by *i *Linker in that, when a subset of members share a haplotype allele, the haplotype allele is maximally extended to the point where the sharing on the particular chromosomal region changes. In other words, the number of chromosomal regions of the same sharing status is minimized in xPedPhase, and so the determined haplotype allele sharing is the most parsimonious. We see this as an advantage over *i *Linker, which only deduces haplotype allele sharing from the greedy haplotyping results. Afterwards, the shared haplotype alleles and their sharing information can readily be used in various association studies [9-11].

## Results

### Breakpoint recovery

In the genotype data simulation process (see "Methods" for details), a simulated parental breakpoint could arise between two consecutive homozygous SNP sites and thus it is not possible to be precisely recovered by any computational haplotyping algorithms. In addition, *i *Linker is a greedy program that introduces a breakpoint only if necessary; and xPedPhase minimizes the number of zero-recombination chromosomal regions. That is, both programs work in certain most parsimonious ways.

We define the following measures of effectiveness for our haplotype allele sharing status determination process. A simulated breakpoint is classified as *recovered *if there is an inferred breakpoint (by *i *Linker or xPedPhase, respectively) that (1) is identical to the simulated breakpoint, or (2) can be "moved" to the simulated breakpoint site [12], *i.e*., the parental SNPs in between these two sites are all homozygous. Subsequently, the breakpoint recovery *precision *is defined as the number of simulated breakpoints being recovered (*true positives*) divided by the number of breakpoints generated by *i *Linker or xPedPhase (true and false positives). The breakpoint recovery *recall *is defined as the number of simulated breakpoints being recovered (true positives) divided by the number of simulated breakpoints (true positives and false negatives).

For each of the 10 distinct pedigrees tested, we used 5 sets of real, unrelated, chromosome 1 genotype data obtained from GeneChip Human Mapping 10 K Xba [49], as well as 5 sets from 50 K Xba [12] arrays to assign haplotypes for founders, by randomly specifying the paternal and the maternal SNP alleles at the heterozygous sites. There were, in total, 877 and 4, 658 SNP sites in the 10 K and 50 K data, respectively. For each set (and each pedigree), we simulated 10 instances. The breakpoint recovery precision and recall associated with each pedigree are computed as the averages over the 50 corresponding instances. These values on the 10 K and 50 K data are collected in Tables 1 and 2, respectively, where pedigree *n*_1_-*n*_2 _[-*n*_3_] has *n*1(*n*_2_, [*n*_3_,] respectively) members in the first (second, [third,] respectively) generation. The overall breakpoint recovery precision and recall by *i *Linker, averaged over 500 10 K simulated instances, were98.4% and 96.4%, respectively; The corresponding ones by xPedPhase were 91.2% and 97.8%, respectively. On 500 50 K instances, xPedPhase were not able to return results for pedigrees 2-2 and 2–3 (see "Discussion" for some explanations); Its overall breakpoint recovery precision and recall, averaged over 400 50 K simulated instances, were 95.7% and 98.3%, respectively. *i *Linker finished all of the 50 K instances, achieving overall breakpoint recovery precision and recall of 99.6% and 97.2%, respectively.

**Table 1:** 10 K breakpoint recovery results

	*i *Linker		xPedPhase		Block-Extension	
	
Pedigree	Precision	Recall	Precision	Recall	Precision	Recall
2-2	0.994	0.936	0.971	0.952	0.253	1.000

2–3	0.982	0.965	0.964	0.966	0.326	0.999

2-3-1	0.985	0.965	0.961	0.972	0.214	0.999

2-3-2	0.989	0.962	0.955	0.972	0.151	0.995

2-3-3	0.972	0.968	0.935	0.976	0.177	0.996

2-3-5	0.977	0.971	0.872	0.989	0.160	0.997

2-4-3	0.984	0.969	0.924	0.978	0.203	0.999

2-5-4	0.989	0.949	0.882	0.976	0.231	0.999

2-5-5	0.991	0.970	0.846	0.989	0.204	0.998

2-6-5	0.986	0.956	0.867	0.984	0.212	0.999

Average	0.984	0.964	0.912	0.978	0.213	0.998

Breakpoint recovery results: Average breakpoint recovery precision and recall by *i *Linker, xPedPhase, and Block-Extension on 10 K genotype datasets.

**Table 2:** 50 K breakpoint recovery results

	*i *Linker		xPedPhase	
	
Pedigree	Precision	Recall	Precision	Recall
2-2	1.000	0.967	-	-

2–3	0.994	0.969	-	-

2-3-1	1.000	0.971	0.977	0.978

2-3-2	1.000	0.976	0.986	0.981

2-3-3	0.991	0.981	0.969	0.988

2-3-5	0.993	0.973	0.950	0.987

2-4-3	0.992	0.976	0.966	0.981

2-5-4	0.996	0.966	0.932	0.985

2-5-5	0.996	0.965	0.937	0.982

2-6-5	0.997	0.972	0.942	0.983

Average	0.996	0.972	0.957	0.983

Breakpoint recovery results: Average breakpoint recovery precision and recall by *i *Linker and xPedPhase on 50 K genotype datasets.

We also collected the breakpoint recovery results by the Block-Extension algorithm inside the PedPhase package [50]. Due to the fact that it has a limit on the number of SNPs, Block-Extension was ran only on the 500 simulated 10 K genotype datasets. Overall its running time was very similar to *i *Linker, that both programs ran in seconds. Its precision and recall are collected in Table 1, with the average breakpoint recovery precision 21.3% and recall 99.8%. An interesting observation is that the Block-Extension algorithm always generated about 5 times more breakpoints than the simulated ones, which is one of the main causes for low precisions.

### Haplotype allele sharing recovery

For each simulated pedigree genotype dataset, the simulated mutation region was recorded (for the case-control association study; such a region is shared by all diseased members but no healthy members). We also recorded all the chromosomal regions that are exclusively shared by all the diseased members in the simulated haplotype dataset, before transforming it into the genotype dataset. We call them the *simulated shared regions*. Note that the simulated mutation region is always contained in one of the simulated shared regions. After running *i *Linker and xPedPhase, based on the reported haplotype allele sharing, we identify all the chromosomal regions that are shared by all and only diseased members too, which we call the *discovered shared regions*. If the discovered shared regions contain the simulated mutation region, then the simulated mutation region is *recovered*.

Among the 500 simulated 10 K genotype datasets, xPedPhase missed 14 simulated mutation regions, 10 of which are among the 23 ones missed by *i *Linker. That is, the simulated mutation region recovery accuracies by xPedPhase and *i *Linker were 97.2% and 95.4%, respectively. The Block-Extension algorithm missed 102 simulated mutation regions, achieving a significantly lower accuracy of 79.60%. For the 400 simulated 50 K genotype datasets, xPedPhase missed only 4 simulated mutation regions; *i *Linker missed 2 additional ones but it recovered 100 more instances; These gave xPedPhase and *i *Linker the simulated mutation region recovery accuracies of 99.0% and 98.8%, respectively.

**Figure 1:**
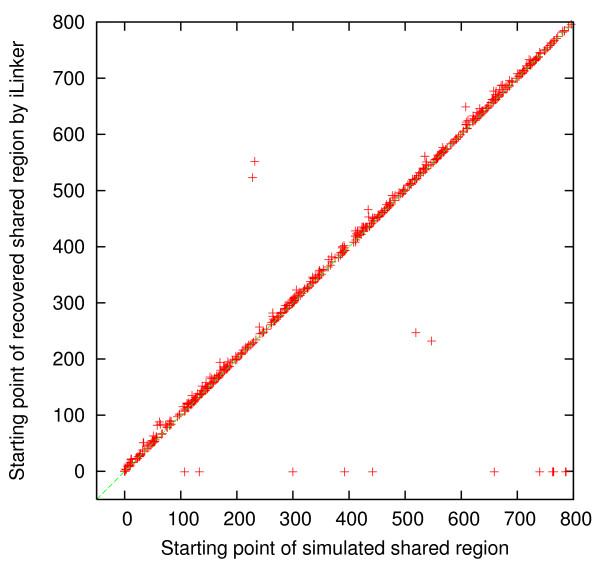
**Scatter plot of the starting SNP sites of shared regions: simulated vs. discovered by *i *Linker on 500 simulated 10 K genotype datasets**.

**Figure 2:**
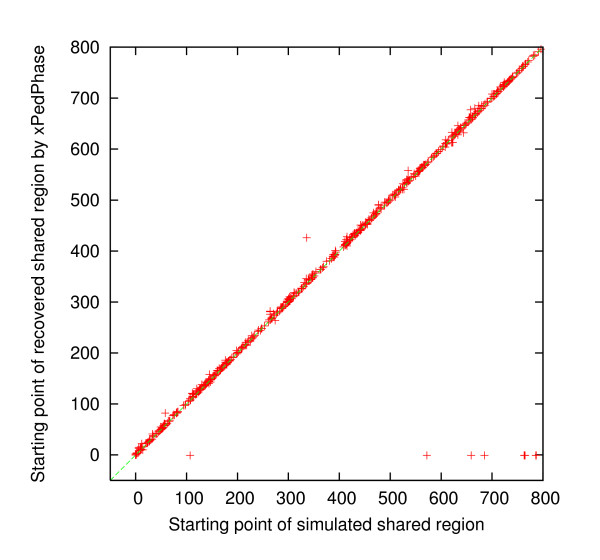
**Scatter plot of the starting SNP sites of shared regions: simulated vs. discovered by xPedPhase on 500 simulated 10 K genotype datasets**.

**Figure 3:**
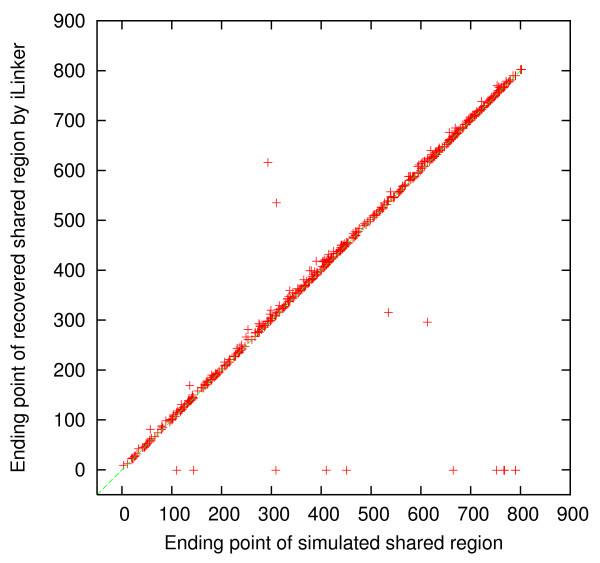
**Scatter plot of the ending SNP sites of shared regions: simulated vs. discovered by *i *Linker on 500 simulated 10 K genotype datasets**.

**Figure 4:**
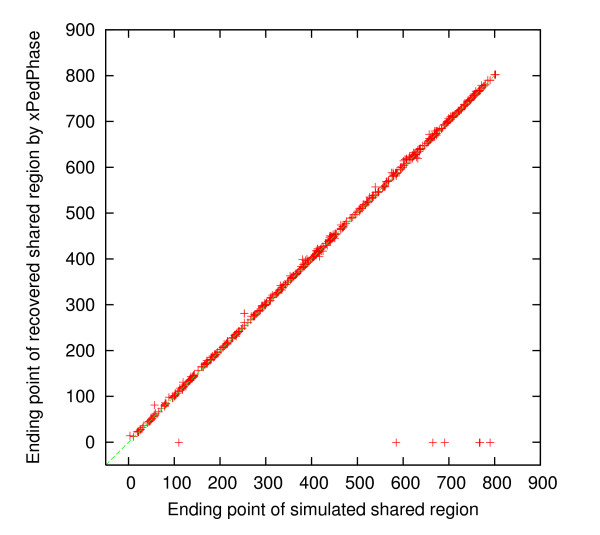
**Scatter plot of the ending SNP sites of shared regions: simulated vs. discovered by xPedPhase on 500 simulated 10 K genotype datasets**.

For each simulated dataset, we compared the simulated shared regions and the discovered shared regions, by *i *Linker and xPedPhase separately, to determine whether or not each simulated shared region was recovered by checking if they overlap or not. If a simulated shared region was not recovered, then the corresponding discovered region was set to [-1, -1]. For the 500 simulated 10 K genotype datasets, there were 725 simulated shared regions in total. 7 of them were not recovered by either xPedPhase or *i *Linker; 2 additional were not recovered by xPedPhase and 5 additional were not recovered by *i *Linker. We collected the starting SNP site and the ending SNP site for each of these simulated shared regions (*x*-axis) and those for the corresponding discovered shared region (*y*-axis) by xPedPhase and *i *Linker, respectively, and plotted them in Figures 1, 2, 3 and 4. Essentially, these plots show the extent to which the discovered shared regions are off the simulated shared regions. The correlation coefficients between the two sets of starting and ending sites of recovered shared regions were 0.99981 and 0.99989 by xPedPhase, and 0.99980 and 0.99981 by *i *Linker. On 400 50 K datasets that both *i *Linker and xPedPhase finished, every shared region was recovered by xPedPhase and *i *Linker missed only two. The correlation coefficients were 0.999993 and 0.999928 by xPedPhase, and 0.999988 and 0.999983 by *i *Linker, respectively (the scatter plots are not included since they are basically straight lines).

## Discussion

### *i *Linker vs. xPedPhase: breakpoint recovery

Both *i *Linker and xPedPhase determine the haplotype allele sharing status among pedigree members through *partial *haplotyping. That is, *i *Linker repeatedly runs a greedy haplotyping algorithm on the smallest nuclear families in the pedigree, with an objective function designed to minimize the total number of breakpoints. xPedPhase, on the other hand, repeatedly searches for the maximal zero-recombination chromosomal regions along the chromosome, and thus minimizes the total number of breakpoint sites. Both programs therefore determine the haplotype allele sharing in certain most parsimonious ways.

From our extensive simulation study, we found that xPedPhase generated slightly more breakpoints per meiosis than *i *Linker. For example, on average, the number of simulated breakpoints per meiosis (disregarding male and female difference) was 2.38, and the number of breakpoints per meiosis generated by *i *Linker was 2.30, which is slightly less, likely due to its greedy nature. But the number of breakpoints per meiosis generated by xPedPhase was 2.76, greater than 10% more than simulated. Nevertheless, among these 2.76 breakpoints per meiosis by xPedPhase, 2.35 were actually true positives; while among the 2.30 breakpoints per meiosis by *i *Linker, 2.27 were true positives. This explains the slightly higher recalls by xPedPhase than *i *Linker – xPedPhase introduced a few more breakpoints, of which some were true positives though the others were false positives. Also interestingly, for almost all instances, the numbers of breakpoint sites by xPedPhase were equal to the numbers of breakpoints by *i *Linker. This fact explains why the correlation coefficients of starting (and ending, respectively) SNP sites between the simulated shared regions and the shared regions discovered by xPedPhase and *i *Linker are nearly identical.

### Mutation region recovery

Among the 500 simulated 10 K genotype datasets, xPedPhase missed 14 simulated mutation regions and *i *Linker missed 23. They both missed the simulated mutation region on 10 datasets. (On the other hand, the Block-Extension algorithm missed 102, a much larger number of, simulated shared regions.) We carefully examined these 10 datasets and found out a common pattern of the simulated mutation regions. These simulated mutation regions were short, containing only 2 to 4 SNPs, and the specified diseased haplotype allele was not unique, *i.e*., when this allele was paternal, there was a maternal allele exactly the same. Such a phenomenon is caused by our simulation process, which does not do the checking for uniqueness. The consequence is that, the diseased allele was not exclusively found in diseased members but rather, it was shared among some healthy members. Therefore, none of *i *Linker and xPedPhase were able to recover it.

### SNP density

From the simulation study results, one can see that, using higher density SNP makers, 50 K over 10 K in our case, both *i *Linker and xPedPhase performed better, in terms of the breakpoint recovery and the haplotype allele sharing recovery. In particular, on 50 K genotype datasets, the discovered shared chromosomal regions exclusive to all the diseased members were almost identical to the simulated shared regions, achieving all higher than 0.999 correlation coefficients. This fact is certainly desirable in the case-control association studies. On the other hand, both *i *Linker and xPedPhase also performed very well on 10 K genotype datasets. This hints that they can be useful in haplotype-based association studies on species, such as cattle [51] and soybean [52], for which no high but only medium density SNP arrays are available.

### *i *Linker vs. xPedPhase: running time

The zero-recombination haplotyping algorithm inside PedPhase runs in *O*(*m*^3^*n*^3^) time, where *m *is the number of SNP makers and *n *is the size of the pedigree. xPedPhase thus needs cubic time as well on each maximal zero-recombination chromosomal region. For the pedigrees used in the simulation study, preliminary testing using a zero-recombination segment of more than 600 SNPs caused the program to either crash or run for hours. In the final batch run of the programs to collect results, several restarts were required on simple pedigrees such as 2-2 and 2–3, even on 10 K instances. Program *i *Linker did not have the running time issue, where it always returned a solution within seconds. xPedPhase, on the other hand, could not return results on most of the 50 K instances for pedigrees 2-2 and 2–3. Therefore, the collected results for xPedPhase on 50 K data are only for 8 pedigrees.

Given that xPedPhase, which non-trivially employs the cubic time zero-recombination haplotyping algorithm, could run for hours on small pedigrees, part of our future attention is to implement the linear time algorithm by Liu and Jiang [45], which does the same zero-recombination haplotyping but was described as difficult to implement. Eventually, one might want to design a novel linear time zero-recombination haplotyping algorithm that, similarly in one whole genome scan, determines all maximal zero-recombination chromosomal regions, together with their zero-recombination haplotyping solutions.

### Choice of *i *Linker or xPedPhase

*i *Linker performed better in terms of breakpoint recovery precision, but it had slightly lower recalls than xPedPhase. xPedPhase seemingly generated more breakpoints, some of which picked up the simulated ones. Both *i *Linker and xPedPhase have the algorithmic nature to push breakpoints to the end of the chromosome, which is validated from the scatter plots of the starting and ending SNP sites of the discovered shared regions versus the simulated ones (Figures 1, 2, 3, 4). Also, in terms of shared region recovery, xPedPhase did better than *i *Linker, possibly due to the more breakpoints it generated.

*i *Linker accepts datasets containing genotype errors, and it has a step to correct not only those sites violating Mendelian inheritance rules but also pairs of unlikely close breakpoints, in terms of their physical distance. In fact, there were instances in which two adjacent breakpoints on a member are less than one million basepairs apart, and *i *Linker smoothed the region by revising the genotype data. This is another reason that *i *Linker missed several very short simulated shared regions. Note that PedPhase, and consequently xPedPhase, does not tolerate any genotype errors, neither missing data.

On each of the 1, 000 instances, disregarding the SNP density, *i *Linker ran in only seconds. The running time of xPedPhase varied a lot, from seconds to minutes to hours. Given a pedigree, the running time of xPedPhase is determined by the length of the chromosomal region under consideration. When the pedigree is not too small, the lengths of zero-recombination chromosomal regions were only tens or hundreds and xPedPhase was able to deal with them in seconds to minutes too. For pedigrees 2-2 and 2–3, such lengths could be thousands. We had waited for days without the complete results and thus terminated xPedPhase. In summary, xPedPhase performed slightly better, in particular when we do not want to miss many breakpoints, at the cost of longer running time.

### Dealing with missing genotype values in *i *Linker

Note that PedPhase does not tolerate any missing genotype data, neither errors. Consequently, xPedPhase does not work on datasets with missing data or errors. But *i *Linker deals with missing genotype data, by ignoring them during the haplotyping process and then imputing them using the haplotype inheritance. Conceivably, such a way of treating missing genotype data would reduce the haplotyping accuracy. On each of the 1000 simulated genotype datasets, we manually erased a portion of data points, at 0.5%, 1%, 1.5%, 2%, 2.5% and 3% respectively, and then ran *i *Linker to collect its breakpoint recovery and the mutation region recovery. The breakpoint recovery precision and recall are collected in Tables 3 and 4, respectively, for 10 K and 50 K datasets. Clearly seen, while precision remained largely the same, the recall dropped a little with increasing missing rates. Nevertheless, overall these small percentages of missing genotype data did not affect the breakpoint recovery accuracies much. However, the mutation region recovery by *i *Linker could drop a lot when the SNP density is low. For example, 23, 28, 29, 42, 52, 56, 56 simulated mutation regions were missed among the 500 10 K datasets with 0%, 0.5%, 1%, 1.5%, 2%, 2.5%, and 3% missing data points, respectively; while only 6, 11, 9, 12, 9, 11, 10 simulated mutation regions were missed among the 500 50 K datasets, respectively.

**Table 3:** 10 K breakpoint recovery results at the presence of missing data

	0.5%		1%		1.5%		2%		2.5%		3%	
	
Pedigree	Precision	Recall	Precision	Recall	Precision	Recall	Precision	Recall	Precision	Recall	Precision	Recall
2-2	0.998	0.968	0.998	0.926	0.997	0.941	0.998	0.937	0.987	0.946	1.000	0.944

2–3	0.983	0.969	0.997	0.968	0.989	0.970	0.998	0.967	0.993	0.967	0.989	0.948

2-3-1	0.994	0.973	0.994	0.959	0.986	0.959	0.986	0.947	0.988	0.952	0.992	0.968

2-3-2	0.981	0.968	0.981	0.971	0.984	0.947	0.984	0.947	0.985	0.950	0.985	0.945

2-3-3	0.989	0.974	0.967	0.967	0.990	0.962	0.962	0.962	0.969	0.950	0.983	0.958

2-3-5	0.974	0.972	0.966	0.966	0.964	0.960	0.960	0.961	0.970	0.954	0.959	0.951

2-4-3	0.984	0.964	0.985	0.960	0.985	0.953	0.986	0.964	0.978	0.950	0.99	0.956

2-5-4	0.980	0.952	0.984	0.953	0.979	0.950	0.984	0.940	0.983	0.939	0.980	0.932

2-5-5	0.980	0.961	0.971	0.958	0.980	0.947	0.983	0.947	0.982	0.935	0.973	0.930

2-6-5	0.985	0.952	0.987	0.952	0.984	0.939	0.983	0.930	0.982	0.922	0.984	0.911

Average	0.985	0.965	0.983	0.958	0.984	0.950	0.983	0.950	0.982	0.947	0.984	0.944

Breakpoint recovery results: Average breakpoint recovery precision and recall by *i *Linker at the presence of 0.5–3% missing genotype data.

**Table 4:** 50 K breakpoint recovery results at the presence of missing data

	0.5%		1%		1.5%		2%		2.5%		3%	
	
Pedigree	Precision	Recall	Precision	Recall	Precision	Recall	Precision	Recall	Precision	Recall	Precision	Recall
2-2	0.991	0.974	1.000	0.972	1.000	0.972	1.000	0.966	0.996	0.964	1.000	0.964

2–3	0.999	0.982	0.999	0.975	0.996	0.973	0.991	0.976	0.999	0.979	0.998	0.971

2-3-1	0.991	0.975	0.999	0.975	0.996	0.972	0.990	0.976	0.999	0.979	0.998	0.971

2-3-2	0.992	0.972	0.990	0.977	0.995	0.973	0.998	0.960	0.994	0.980	0.995	0.972

2-3-3	0.996	0.977	0.992	0.971	0.996	0.968	0.993	0.969	0.994	0.973	0.994	0.968

2-3-5	0.988	0.973	0.988	0.965	0.990	0.965	0.986	0.969	0.987	0.973	0.982	0.967

2-4-3	0.996	0.981	0.994	0.974	0.997	0.979	0.995	0.969	0.991	0.969	0.991	0.969

2-5-4	0.997	0.974	0.996	0.969	0.980	0.971	0.997	0.966	0.998	0.968	0.996	0.954

2-5-5	0.989	0.977	0.994	0.969	0.996	0.972	0.996	0.968	0.984	0.967	0.992	0.969

2-6-5	0.998	0.976	0.995	0.966	0.997	0.966	0.978	0.962	0.982	0.953	0.992	0.953

Average	0.994	0.976	0.995	0.971	0.994	0.971	0.992	0.968	0.992	0.970	0.994	0.966

Breakpoint recovery results: Average breakpoint recovery precision and recall by *i *Linker at the presence of 0.5–3% missing genotype data.

### Other possible applications

Studies have shown that the human genome can be partitioned into large blocks with high LD and relatively low recombination, separated by short regions of low LD [6-8]. The same things are also expected for other species such as cattle and soybean. In the cattle breeding industry, normally small to medium size pedigrees can be easily collected. By running our haplotype allele sharing status determination programs on these pedigrees, we will be able to locate those crossover sites for each pedigree. These results can thus be used to compose the map of crossover sites and thus identify the *crossover hotspots *along the genome. We expect that our programs will be useful in many genomics selection projects, for example, to provide deterministic haplotype allele sharing and shared haplotype alleles for various *quantitative trait locus *(QTL) identification and quantitative association studies.

## Methods

### Haplotype allele sharing by xPedPhase

PedPhase is a haplotyping program consisting of four algorithms [42]. The constraint-finding algorithm first determines whether a pedigree genotype dataset has zero-recombination haplotype configurations and identifies all such configurations if it does. More precisely, the algorithm first identifies all necessary (and sufficient) constraints on the haplotype configurations derived from the Mendelian inheritance rules and the zero-recombination assumption, represented as a system of linear equations on binary variables over the cyclic group Ƶ_2 _(i.e., integer addition module 2). It then solves the equations to obtain all consistent haplotype configurations satisfying the constraints, using a simple method based on Gaussian elimination. These consistent haplotype configurations are shown to be feasible zero-recombination solutions. The running time for representing and solving the equations is *O*(*m*^3^*n*^3^), where *m *is the number of SNPs under consideration and *n *is the number of non-founder members in the pedigree, and the time for enumerating all configurations is proportional to the number of feasible zero-recombination solutions.

This cubic running time is due to the Gaussian elimination procedure that is employed to solve a system of (at most) 2(*m *- 1)*n *linear equations. When considering one more SNP site, the increase in the number of equations is at most 2*n*. This observation leads to our extension of the PedPhase, denoted as xPedPhase, for one whole genome scan to determine all maximal zero-recombination chromosomal regions. Let *M *denote the total number of SNPs on the chromosome under consideration. We first run PedPhase on the foremost two SNPs. If the Gaussian elimination procedure reaches no solution, then there is a breakpoint in between these two sites, and we proceed to run PedPhase on the second and the third SNPs. Otherwise, generate (at most) 2*n *linear equations by considering the last SNP and the next SNP which has not been examined. Append these new linear equations to the existing reduced system, and continue applying the Gaussian elimination procedure. Again, if the procedure reaches no solution, then there is a breakpoint in between the last two SNP sites, and we proceed to run PedPhase on the last SNP and the next SNP which has not been examined. Otherwise, generate (at most) 2*n *linear equations by considering the last SNP and the next SNP which has not been examined, and so on.

Note that in order to output the haplotype configurations for each maximal zero-recombination chromosomal region, we need to save the last set of solutions before we consider a new SNP site. xPedPhase thus runs in *O*(*M*^3^*n*^3^) time, where *M *denotes the total number of SNPs. In the haplotype configuration for each maximal zero-recombination chromosomal region, the haplotype alleles of the founders are carefully swapped so that the total number of breakpoints is minimized. For this purpose, we set the rule as that, for each pair of founders, their haplotype alleles on two consecutive maximal zero-recombination chromosomal regions are such that the total number of breakpoints in their children is no more than half the number of their children. On almost all maximal zero-recombination chromosomal regions identified in our extensive simulation experiments, xPedPhase returned a unique solution. In the case of multiple solutions, we chose to use the first solution returned from xPedPhase, though ideally we would check for the one that results in the minimum number of breakpoints. After the haplotype alleles for each member have been determined, we report the sharing information among all the pedigree members.

### Haplotype allele sharing by *i *Linker

*i *Linker determines the haplotype allele sharing status for individuals in a pedigree [12]. The key component of this program is a rule-based and greedy haplotype inference algorithm that assigns haplotypes to the *smallest nuclear families *extracted from the pedigree. A smallest nuclear family is either a trio, or one parent and a child. *i *Linker traverses the pedigree in a top-down fashion, and determines haplotypes of family members in sequence. Overall, the program tries to use a minimum number of breakpoints to explain the pedigree genotype data. During the genotype data interpretation process, parental haplotype phases can be revised when more members are added, as long as the revision reduces the total number of breakpoints and still explains the genotype data. Additionally, *i *Linker has an error correction step that detects unlikely crossover events.

It is worth noting that *i *Linker is the first to emphasize correctly inferring allele sharing status (rather than haplotype phases) among pedigree members. This greatly reduces computational complexity, even in the face of large high-density SNP genotype datasets. *i *Linker has been extremely successful in both breakpoint recovery and identifying linked regions for case-control association studies [12].

### Genotype data simulation

In this study, we have implemented a simulation process which generates the haplotypes for a child using the parents' haplotypes according to the *χ*^2^(*m*)-model for crossover events with *m *= 4 [53,54]. Using this trio generation process as a basis, genotype datasets for large pedigrees can be simulated for testing our programs. We note that this genotype data generation process slightly differs from what has been done in Lin *et al*. [12], and differs completely from several existing genotype/haplotype data generation programs such as Simlink [55], Simulate [56], Ilink in the Fastlink/Linkage package [57], Slink [32], Allegro [58], Merlin [59], Simla [60], and SimPed [61]. In many of these programs the crossover events are simulated according the probabilities specified for every two adjacent marker sites. In the case of high density SNP markers, all these probabilities are tiny (e.g., from the International HapMap Project [22]).

In our genotype data simulation process, the input to the trio generation process consists of the haplotypes (for one chromosome) for both parents, the physical loci for all the SNP markers, the genetic map corresponding to the chromosome (from the International HapMap Project [22]), and the average numbers of crossovers on the chromosome among the female and male population, respectively.

The children haplotypes are generated through random inheritance of parental alleles after simulating crossover events. The *χ*^2^(*m*)-model assumes that crossover intermediates (C events) are distributed along the four-strand sister chromatid bundle with a rate of 2(*m *+ 1) C events per chromosomal interval, and every C event resolves in either a crossover (Cx) or not (Co). Furthermore, when a C event resolves in a Cx, the next *m *C events must resolve as Co's, followed by another Cx event, i.e. the C events resolve in a sequence of ... Cx(Co)^*m*^Cx(Co)^*m*^... The leftmost C event has an equal chance to be one of Cx(Co)^*m *^[53]. The simulation process determines the chromosomal intervals by reading, from the genetic map, the physical loci for all the SNP markers, and the average numbers of crossovers. It then divides the whole chromosome from head to tail such that the length of each interval (except the last one) is equal to the genetic distance (in Morgans) required for one crossover. For human chromosome 1, this genetic distance is about 1.7 Morgans for males and 0.9 Morgans for females, respectively. The simulation process assumes no chromatid interference, and the child is simulated to randomly inherit one strand of the four-strand chromatid bundle from each parent.

To generate a pedigree genotype dataset, the simulation process locates the individual(s) both of whose parents' haplotypes are either known or have been simulated, and then generates its haplotypes. It then locates the siblings who have only one parent in the pedigree, where its haplotypes are either known or have been simulated, randomly generates the haplotypes for the other parent, and then generates all the children haplotypes one by one. Finally, when all individuals' haplotypes have been simulated, the allele parental information is erased at each SNP site, to give the genotype data.

To validate that the haplotype allele sharing status can be correctly recovered by our programs, we simulate the pedigree genotype data for case-control association study. Before the simulation process, a mutation region of length in between 0 and 10 Mbps, and containing at least one heterozygous site, is randomly assigned to be close to a SNP site in one haplotype of one of the founders. Then, during the simulation process, the affected offspring are forced to inherit one of the mutation strands (there are exactly two among the four strands) and the unaffected are forced not to inherit any of the mutation strands. Note that when a Cx event cuts into the mutation region, then it has to be pushed to the last Co event overlapping with the mutation region. That is, the affected offspring should inherit the complete mutation region.

Since pedigrees of two or three generations are the most common in our previous experience with human disease association studies and more recent experience in cattle breeding industry, we used 10 pedigrees of two or three generations and of size 4 to 13 in the simulation study for validation purpose, in which the disease status for the members are semi-randomly assigned. For each pedigree, we used 5 sets of real unrelated genotype data for the founders; And for each set, we simulated 10 genotype datasets. Those real unrelated genotype data were obtained from two different SNP microarrays, GeneChip Human Mapping 10 K Xba and 50 K Xba arrays. In total, we have simulated 500 10 K and 50 K genotype datasets, respectively.

## Conclusion

We have showed that for pedigree genotype datasets, the haplotype allele sharing status among the members can be deterministically, efficiently, and accurately determined, even for very small pedigrees, by two most parsimonious partial haplotyping methods. Given its excellent performance in both the breakpoint recovery and the shared region recovery, the program can be useful in many applications including haplotype based association studies.

## Authors' contributions

GL conceived the overall project. ZC and GL detailed the methods. ZC, HS, JX, and YW performed all the experiments, and all authors were involved in the result interpretation and discussion. GL drafted, and finalized the manuscript with RG and PS. All authors read and approved the final manuscript.
